# Effects of two different dual-task training protocols on gait, balance, and cognitive function in community-dwelling older adults: a 24-week randomized controlled trial

**DOI:** 10.7717/peerj.15030

**Published:** 2023-04-21

**Authors:** Francis Trombini-Souza, Vitória Thaysa Gomes de Moura, Lucas Willian Nunes da Silva, Iara dos Santos Leal, Cleber Anderson Nascimento, Paloma Sthefane Teles Silva, Monica Rodrigues Perracini, Isabel CN Sacco, Rodrigo Cappato de Araújo, Marcelo de Maio Nascimento

**Affiliations:** 1 Department of Physical Therapy, University of Pernambuco, Petrolina, Pernambuco, Brazil; 2 Master’s and Doctoral Programs in Rehabilitation and Functional Performance, University of Pernambuco, Petrolina, Pernambuco, Brazil; 3 Master’s and Doctoral Programs in Physical Therapy, Universidade Cidade de São Paulo, São Paulo, São Paulo, Brazil; 4 School of Medicine, Department of Physical Therapy, Universidade de São Paulo, São Paulo, Brazil; 5 Department of Physical Education, Federal University of São Francisco Valley, Petrolina, Pernambuco, Brazil

**Keywords:** Dual task, Gait, Cognition, Postural balance

## Abstract

**Background:**

Although alternating dual-task (ADT) training is functionally easier for older adults, a large part of the motor and cognitive tasks is simultaneously performed, especially during activities of daily living that require maintaining body balance.

**Objective:**

To evaluate the effects of mixed dual-task training on mobility, cognitive function, and balance in community-dwelling older adults.

**Methods:**

Sixty participants were randomly allocated at a 1:1 ratio into the experimental group—single motor task (SMT) and simultaneous dual task (SDT) interchangeably in stage 1 (for 12 weeks) and after strictly with SDT in stage 2 (the last 12 weeks)—or into the control group—only SMT and SDT interchangeably in stages 1 and 2. Gait parameters were acquired by two inertial sensors. Physical and cognitive performance were acquired by specific questionnaires. Generalized linear mixed models were used for analyzing interaction and main effects.

**Results:**

No between-group difference was observed for gait performance. Both protocols improved mobility (mean change ((MC) = 0.74)), dual-task effect (MC = −13.50), lower limb function (MC = 4.44), static (MC = −0.61), and dynamic balance (MC = −0.23), body sway (MC = 4.80), and cognitive function (MC = 41.69).

**Conclusion:**

Both dual-task training protocols improved these outcomes.

## Introduction

Some cognitive skills, such as processing speed, memory, visuospatial, and executive function abilities, are linked with natural aging (Harada, Natelson Love & Triebel, 2013). Decreases in cortical thickness and volume, reduction of the gray matter, hippocampus and cerebellum constriction, neuronal atrophy, spinal narrowing, synapse reductions, and some traditional biological signaling pathways are all potential contributors to aging-related cognitive impairment (Bettio, Rajendran & Gil-Mohapel, 2017; Harada, Natelson Love & Triebel, 2013; Raz et al., 2005; Storsve et al., 2014). Both cognitive and motor functions are simultaneously controlled by some brain regions such as the frontal lobes, cerebellum, and basal ganglia, which work together to exert governance and control over executive function and the intentionality of motions that entail anticipation and the prediction of other people’s movements (Leisman, Moustafa & Shafir, 2016).

Thus, as people age, their ability to manage dual-task tasks becomes limited (Manor et al., 2018), since the cognitive overloading demanded by the dual-task paradigm is larger than when simply a motor or cognitive task is performed independently. With aging, the central nervous system becomes less and less capable of properly processing a greater volume of sensorial inputs coming from different sources at the same time (Menant et al., 2014), which can cause a delay in functional motor strategies and, as a result, increase the risk of accidental falls (Plummer et al., 2015; Silsupadol et al., 2009b). Thus, dual-task training benefits have been better than single-task approaches (Silsupadol et al., 2009b). Usually, dual-task demands have included mental tracking (serial subtractions), verbal fluency (identifying animal kinds aloud), and manual motor activity (*e.g*., carrying a glass of water on a tray with one hand) (Hunter et al., 2018).

Regarding walking, cognitive-motor dual-task demand, such as walking and talking or walking and solving mathematical calculations (Yuan et al., 2015), compromises gait speed (Smith et al., 2017), stride length, double support phase (Dingwell, Salinas & Cusumano, 2017), and postural control in older adults (Ghai, Ghai & Effenberg, 2017; Pieruccini-Faria et al., 2019). Although the ability to prioritize and allocate attention between two or more tasks becomes progressively compromised throughout aging (Bergamin et al., 2014), dual-task training has the potential to improve the ability of older adults to share attention between motor and cognitive tasks (Wollesen et al., 2019) or additional motor tasks such as using one hand to hold a glass of water on a tray while walking (Hunter et al., 2018). This type of demand generates greater challenges to the cognitive skills of older adults (Lauenroth, Ioannidis & Teichmann, 2016), and consequently increases the recruitment of cognitive resources (Lussier, Bugaiska & Bherer, 2017). Motor and cognitive tasks can be simultaneously performed—when a concurrent attentional focus is required for both activities—or under alternating priority—when the focus of attention alternates between activities (Azadian et al., 2016; Buragada et al., 2012).

In alternating dual-task (ADT) training protocols, participants are usually asked to sometimes focus attention on motor and at times on cognitive task performance (Goh, Pearce & Vas, 2021; Silsupadol et al., 2009a, 2009b). However, it might be challenging to ensure that participants efficiently switch their attention (focus) between motor physical and cognitive tasks. Thus, it seems reasonable to propose a training protocol in which participants sometimes perform a simultaneous dual task (SDT), such as walking and performing mathematical operations, and at times a single motor task (SMT), such as just walking. In this type of protocol, it is possible to ensure, both visually and audibly, that participants are indeed alternating the type of task, *i.e*., sometimes performing only SMT and other times only SDT.

Furthermore, although ADT training has resulted in greater functional performance in older adults, both ADT and SDT have a promising effect on functional performance (Buragada et al., 2012; Ghai, Ghai & Effenberg, 2017; Silsupadol et al., 2009b). When participants are trained under ADT they can learn faster and retain the performance better than under SDT since SDT demands greater complexity of motor and cognitive performance than ADT (Buragada et al., 2012). Thus, ADT may be considered easier (less complex) than SDT. However, it is important to highlight that a large part of the daily motor and cognitive tasks is simultaneously performed, especially during activities that require maintaining body balance in domestic activities (Wang et al., 2015), and in activities such as walking and talking or walking and solving mathematical calculations. SDT are also demanded in activities such as walking while talking to someone, walking through the supermarket and looking for a specific product, or carrying a tray with food while walking (Hofheinz, Mibs & Elsner, 2016).

Thus, we aimed to compare the effects of a mixed dual-task protocol training in which participants underwent initially SMT and SDT interchangeably and progressed strictly with SDT regarding a control protocol in which participants only performed SMT and SDT activities alternately. Considering the experimental protocol will focus especially on tasks that simultaneously require the motor and cognitive interaction, we hypothesized the participants of this training protocol will improve gait performance, functional mobility, static and dynamic body balance, and cognition function over 24 weeks. With this work, we hope to provide clinical practice professionals with a novel dual-task training protocol that moves from simpler dual-task activities—alternating between SDT and ST—to more challenging dual-task activities, performing only simultaneous dual task.

## Materials and Methods

### Experimental design

A randomized controlled clinical trial, with parallel arms and blinding of the evaluator and participants concerning the allocation. A total of 60 participants of both sexes, aged between 60 and 80 years old, were randomly allocated at a 1:1 ratio into an experimental group (EG) or a control group (CG). The participants of the EG underwent a mixed dual-task protocol training in which they performed initially SMT and SDT interchangeably during the first 12 weeks and after they progressed strictly with SDT, in the last 12 weeks of this protocol. The participants of the CG performed only SMT and SDT activities alternately throughout all 24 weeks. Both groups were assessed at allocation (T1), at 24 weeks post-allocation (T2), and after 24 weeks of intervention (T3).

The recommendations of the Declaration of the World Medical Association of Helsinki and the Consolidated Standards of Reporting Trials (CONSORT) were met. The study was approved by the Research Ethics Committee of the University of Pernambuco (study# 2.415.658; CAAE: 71192017.0.0000.5207) and registered prospectively with ClinicalTrial.gov (NCT03886805).

### Sample size

Gait speed during the execution of SDT (primary outcome) was used for sample calculation (Silsupadol et al., 2009a). A minimal clinically important difference of 0.05 m/s was adopted, an effect size of 0.20 (Perera et al., 2006), power of 95% (1 - β), alpha of 5%, and drawing of *F* statistics of repeated measures. Thus, a total of 48 participants were calculated. Considering a sample loss of 20%, 60 participants were assessed and allocated to both groups with a 1:1 ratio. The sample size was calculated using G * Power 3 (Faul et al., 2007).

### Eligibility criteria and recruitment

Older adults who scored ≥ 52 points (up to a maximum of 56 points) on the Berg Balance Scale (Silsupadol et al., 2009a), ≥ 24 points (up to a maximum of 30 points) on the Mini-Mental State Examination (Azadian et al., 2016), and those capable of uninterruptedly walking for a distance of 60 m at a self-selected speed of at least 1 m/s without the help of another person, cane or walker were included. Potential participants were excluded if: (i) they had any contraindications for postural balance and/or cognition training, (ii) had fallen two or more times in the past 12 months, (iii) had enrolled or had participated in any regular and structured physical and cognitive exercise program training, two or more times a week in the last six months, (iv) had any chronic health condition for which physical exercise was contraindicated, and (v) had any upper or lower limb fractures in the last six months. Participants with a diagnosis of uncontrolled diabetes, surgical operation on their knees, ankles, or hips, or recent muscular injuries were also excluded. There was no need to alter the procedures once the study started.

The participants were recruited from the municipality’s primary healthcare units, parks, squares, and churches (Petrolina, PE, Northeast Brazil). The study was publicized through radio commercials, local news, Facebook and Instagram. After being told of the study’s goals and consenting to them, each participant signed an informed consent form.

### Intervention

Participants in each group (EG and CG) attended at least 75% of the training sessions, twice a week, 60 min each, for 24 weeks, which included: (i) a warm-up phase (10 min) with supervised walking on a flat surface, stretching exercises, and joint mobilization; (ii) gait and balance training (four stations, 10 min each, a total of 40 min); and (iii) a relaxation phase, including breathing exercises and global muscle stretching (10 min) (Trombini-Souza et al., 2020). We set up a circuit composed of hula hoops, ropes (in a straight line and zigzagging), an agility ladder, traffic cones, steps, cardboard boxes, and other obstacles arranged on the floor (stable surface) or mattresses (unstable surface) for the protocol of both groups, depending on the aim of each training stage (Trombini-Souza et al., 2020).

**Figure 1:**
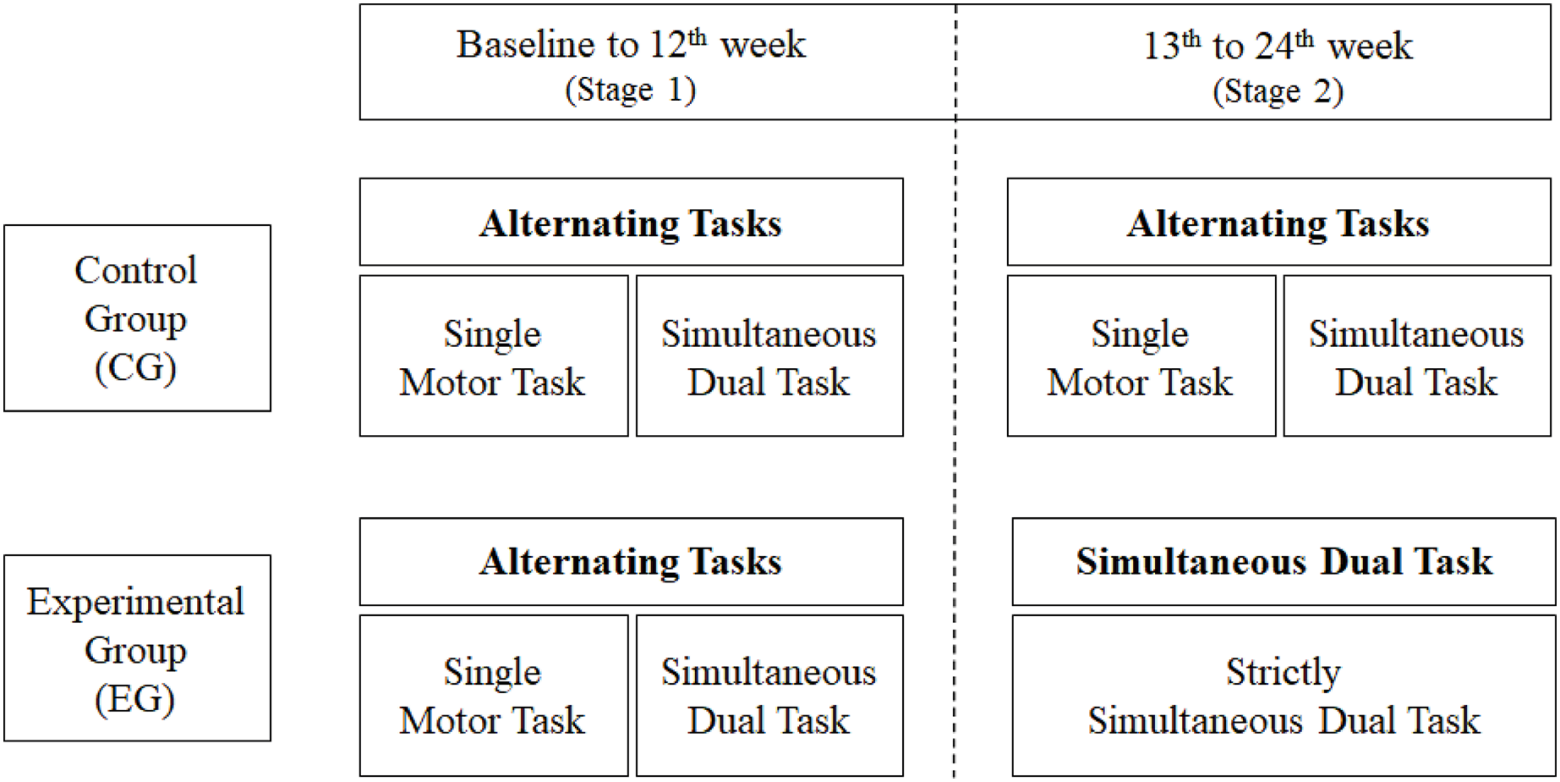
Flowchart of the experimental and control protocols.

Participants of the CG performed a training protocol consisting of alternating between SDT and SMT every 10 min in stages 1 (the first 12 weeks) and 2 (the last 12 weeks) of the training. This interchanging of the tasks was named in this study as alternating tasks protocol. In the SDT component, participants were asked to simultaneously perform motor tasks—such as walking on stable and unstable surfaces, in a straight line, zigzagging, kicking a ball, going over obstacles, and contouring traffic cones—, and cognitive tasks—such as performing mathematical operations, remembering a list of names, and spelling words. At the beginning of the protocol, participants were instructed to walk at their usual gait speed, in a straight line, on a stable surface. With the evolution of the protocol, the participants were asked to increase their gait speed, walk on unstable surfaces (mattresses), on ropes arranged on the floor in a zigzag way, walk and kick a ball, go over obstacles, go around traffic cones, *etc*. In the SMT component, the participants were asked to perform only the motor tasks proposed in the SDT component. On the other hand, the participants of the EG started the dual-task training like the CG, **i.e*.*, alternating SDT and SMT, however, they continued performing only SDT in stage 2. We named this sequencing ‘mixed protocol’, as shown in Fig. 1. A personalized intervention was not planned for this protocol, considering the participants’ independent functional performance; however, minor exercises adaptations were made, when necessary, until the participant could do the exercise without difficulty. The detailed protocol with its evolution in training intensity and volume has been published (Trombini-Souza et al., 2020).

All the protocol training was conducted in a group with a maximum of 15 participants. The interventions were supervised by a physiotherapist with at least 5 years of dual-task training experience, assisted by three students from the last year of the undergraduate course in physiotherapy. Four instructors were responsible for each of the four training stations with a maximum number of four participants. The training protocols were performed in a gym room (20 × 20 m) at the University of São Francisco Valley—UNIVASF.

### Primary outcome

The primary outcome was self-selected gait speed under SDT (Silsupadol et al., 2009a).

Two inertial sensors (Physilog^®5^, Gait Up, Lausanne, Switzerland, Europe) formed by a tri-axial accelerometer (MMA7341LT, range ± 3 g, Freescale, Austin, TX, USA), a tri-axial gyroscope (ADXRS, range ± 600°/s, Analog Devices, Norwood, MA, USA), a memory unit and a microcontroller were used to acquire the speed and other biomechanical gait variables at 128 Hz. The gait variables were collected and imported into a computer, then analyzed using the Gait Analyzer software (Gait Up, Lausanne, Switzerland, Europe).

The sequence of walking conditions was randomly defined before starting the gait acquisition, being (1) conventional walking without cognitive demand, named single task (ST); (2) walking interspersing ST and simultaneous dual tasks (SDT), named alternating task (AT); and (3) walking under SDT. For gait as an ST, the participant was asked to walk a linear distance of 60 m (round trip) at a self-selected walking speed in a straight and flat corridor without performing any cognitive task. For walking under AT, the participant was asked to walk the 60-m course, however, interspersing the ST and SDT (walking and counting back by 3 from the number 100), every 5 m. Participants were asked to continue walking at the same walking speed until the final distance. The command to start and finish the dual task every 5 m was given by the evaluator, according to the traffic cones on the floor placed every 5 m on the floor of the corridor. For walking under SDT, the participant was asked to walk uninterruptedly and count back by 3 s from the number 100 until the 60-m course was completed.

### Secondary outcomes

Secondary outcomes were the gait variability, *i.e.*, the intraindividual standard deviation of gait cycle duration, cycle duration, cadence, stride length, stance and swing phase duration, single and double support duration, and minimum toe clearance (MTC) under all gait conditions, conventional Timed Up and Go (TUG_Conventional_) (Podsiadlo & Richardson, 1991), TUG_Cognitive_ (Woollacott & Shumway-Cook, 2002), postural balance test (Nascimento, Appell & Coriolano, 2012), sit and stand test (from the floor) (Brito et al., 2014), five times sit-to-stand time (5TSTS) (Jones, Rikli & Beam, 1999), forward functional reach test (FFRT) (Duncan et al., 1990), Clinical Test of Sensory Interaction on Balance (CTSIB) (Cohen, Blatchly & Gombash, 1993), versions A and B of the Trail Making Test (TMT-A and TMT-B) (Ricker & Axelrod, 1994), Stroop test (Stroop, 1935), and the dual-task effect (DTE) (Plummer & Eskes, 2015) considering the TUG and the Stroop test, as shown in the following formula.

$\rm{DTE\,(\%) = {\frac{- (dual \;task \;time - single\; task \;time)}{single \;task\; time}}}\times 100$

Dual-task cost or dual-task efficiency is represented by negative and positive values, respectively.

### Randomization and allocation concealment

An independent researcher who was unaware of the representation of the codes assigned to groups produced the randomization code sequence in the WinPepi program (v. 11.65). Following the order generated, the numerical series was preserved in opaque envelopes consecutively numbered from 1 to 60. We kept it secret and kept out of the reach of the participants and evaluators who were blind to the allocation of the sequence of codes until the study’s conclusion.

### Blinding

The evaluators and participants were blinded as to the allocation of individuals. Considering the technical and operational closeness between both training programs, blinding the participants was possible. We confirmed this by asking participants in both groups if they knew which group they had been part of.

### Statistical analysis

**Figure 2:**
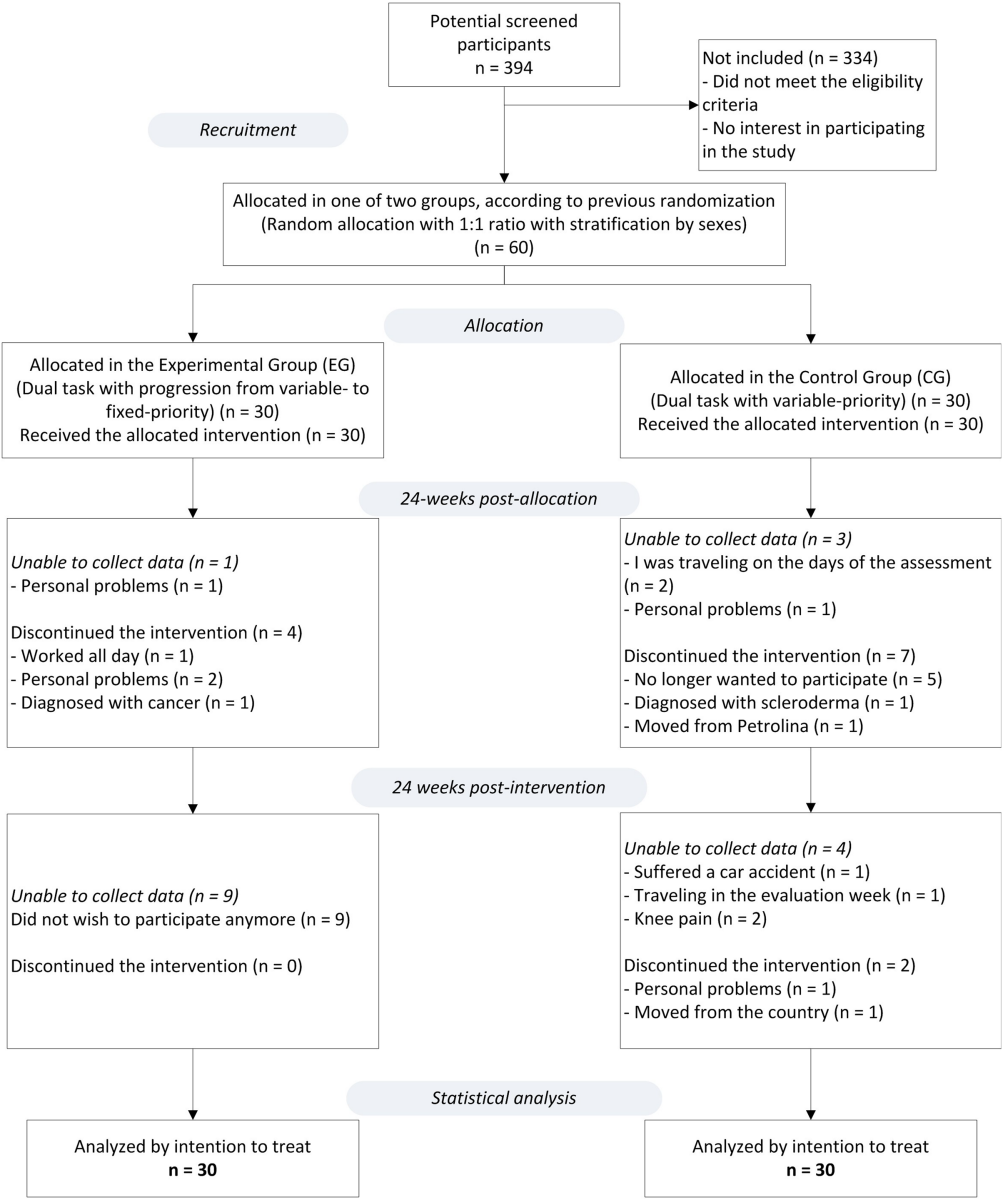
Study flowchart.

Statistical analyzes were performed using the *Statistical Package for the Social Science* (SPSS, IBM; v.22.0), adopting a 5% significance level. The intention-to-treat principle was used. After analyzing the sample missing patterns presented in Fig. 2 (this figure shows the study flowchart) the missing data (study data) were considered to have a completely random cause (missing completely at random—MCAR) and a regression technique for multiple imputations was used. A simple arithmetic mean of 10 imputations was used for each of the study outcomes (Newgard & Haukoos, 2007).

The generalized linear mixed models (GLMM) method was used, considering the group (EG and CG) and time factors (T1, T2, T3), as well as the interaction effect (time *vs*. group). Participants were considered as random effects and time and group as fixed effects. Q-Q graphs were plotted to verify the adequacy (normality) of each model. Adjustments for univariate (main effects) and multivariate (interaction effect) comparisons of the estimated marginal means (EMM) were made using the Bonferroni test. The comparisons between the pairs of EMM were made based on the original scale of each of the dependent variables.

## Results

From a total of 394 potential participants enrolled, only 60 (response rate of 15.2%), of which 52 were female, met the eligibility criteria. Of the 30 participants allocated into each group, 16% (*n* = 5) of the participants of the EG and 33% (*n* = 10) of the CG were not obtained at T2 (after a 24-weeks intervention). Then at T3 (24 weeks after the end of the intervention), 30% (*n* = 9) of the EG and 20% (*n* = 6) of the CG were not obtained. The monitoring of participants took place between March 2019 and March 2020. The reasons for the missing participant data are described in the study flowchart (Fig. 2) and the participants’ features in the baseline are shown in Table 1.

**Table 1:** Demographic, anthropometric and functional characteristics of the experimental and control groups at baseline.

Variable	EG	CG
	Absolute frequency (relative frequency)	
Sex		
Female (*n*; %)	26 (86.6%)	26 (86.6%)
Male (*n*; %)	4 (13.4%)	4 (13.4%)
Fallers		
Yes (*n*; %)	10 (33.0%)	12 (40.0%)
No (*n*; %)	20 (66.0%)	18 (60.0%)
	Mean (standard deviation)	
Age (years)	67 (5)	66 (4)
Body mass (kg)	70.51 (17.31)	68.82 (9.32)
Height (m)	1.55 (0.06)	1.56 (0.07)
Body mass index (kg/m^2^)	24.13 (12.32)	25.14 (10.38)
Simultaneous dual task	Estimated marginal mean (CI 95%)	
Gait speed (m/s)	1.21 [1.15–1.26]	1.26 [1.21–1.32]
Gait variability (%)	3.02 [2.71–3.34]	2.96 [2.64–3.27]
Gait cycle duration (%)	1.02 [1.00–1.04]	1.00 [0.98–1.03]
Cadence (steps/min)	118.23 [115.47–120.99]	120.21 [117.46–122.96]
Stride length (m)	1.20 [1.15–1.24]	1.24 [1.20–1.29]
Stance (%)	58.82 [58.19–59.44]	58.65 [58.03–59.27]
Swing (%)	41.18 [40.56–41.80]	41.34 [40.72–41.96]
Double support (%)	17.51 [16.36–18.67]	17.52 [16.35–18.66]
Minimum toe clearance (cm)	3.20 [2.90–3.40]	3.20 [3.00–3.40]
Alternating task		
Gait speed (m/s)	1.24 [1.19–1.30]	1.29 [1.24–1.34]
Gait variability (%)	2.89 [2.61–3.16]	2.70 [2.43–2.97]
Gait cycle duration (%)	1.00 [0.98–1.03]	0.99 [0.96–1.01]
Cadence (steps/min)	119.57 [116.85–122.28]	121.05 [118.36–123.75]
Stride length (m)	1.21 [1.72–1.25]	1.26 [1.22–1.30]
Stance (%)	58.54 [57.89–59.19]	58.55 [57.90–59.20]
Swing (%)	41.46 [40.72–42.20]	41.44 [40.70–42.17]
Double support (%)	17.03 [15.87–18.19]	17.31 [16.15–18.47]
Minimum toe clearance (cm)	3.30 [3.00–3.60]	3.10 [2.80–3.30]
Single task		
Gait speed (m/s)	1.31 [1.25–1.36]	1.36 [1.31–1.41]
Gait variability (%)	2.71 [2.47–2.94]	2.45 [2.21–2.69]
Gait cycle duration (%)	0.98 [0.95–1.00]	0.97 [0.95–1.00]
Cadence (steps/min)	122.98 [120.19–125.77]	123.78 [121.00–126.68]
Stride length (m)	1.25 [1.25–1.21]	1.29 [1.25–1.33]
Stance (%)	57.91 [57.27–58.55]	58.23 [57.59–58.87]
Swing (%)	42.10 [41.47– 42.73]	41.75 [41.12–42.37]
Double support (%)	15.82 [14.60–17.04]	16.13 [14.91– 17.34]
Minimum toe clearance (cm)	3.30 [3.10–3.60]	3.00 [2.80–3.33]
Timed up & Go (conventional) (s)	10.25 [9.66–10.83]	9.80 [9.22– 10.38]
Timed up & Go (cognitive) (s)	12.96 [12.19–13.74]	12.56 [11.79–13.34]
TUG dual-task effect* (%)	−27.18 [−33.48 to −20.87]	−28.80 [−35.11 to −22.49]
SBER (1 to 3)	0.92 [0.48–1.37]	1.14 [0.70–1.58]
SBIR (1 to 3)	1.69 [1.31–2.07]	1.50 [1.12–1.88]
DBER (1 to 4)	0.22 [0.06 –0.03]	0.11 [−0.44 to 0.26]
DBIR (1 to 4)	0.48 [0.10–0.85]	0.85 [0.47–1.22]
SBEO (1 to 4)	1.70 [1.56–1.83]	1.82 [1.69–1.95]
SBEC (1 to 4)	1.93 [1.79–2.07]	1.85 [1.71–1.99]
SBDO (1 to 4)	2.00 [1.86–2.13]	1.96 [1.82–2.09]
UBEO (1 to 4)	1.96 [1.89– 2.03]	1.96 [1.89–2.03]
UBEC (1 to 4)	2.56 [2.39–2.73]	2.90 [2.73–3.07]
UBDO (1 to 4)	2.70 [2.51–2.89]	2.91 [2.72–3.10]
Total CTSIB (0 to 24)	12.92 [11.35–14.48]	12.94 [11.37–14.51]
5TSTS (s)	14.40 [12.61–16.19]	16.14 [14.35–17.92]
FFRT (m)	16.86 [15.54–18.17]	16.38 [15.07–17.69]
SRT (0 to 10)	3.92 [3.32–4.52]	4.30 [3.71–4.90]
TMT-A (s)	65.00 [54.43–75.56]	73.69 [63.13–84.26]
TMT-B (s)	191.57 [165.90–217.24]	169.73 (144.06–195.41]
Stroop B&W (s)	37.43 [33.86–41.01]	35.69 (32.11–39.26]
Stroop color (s)	83.04 [75.89–90.18]	88.72 [81.57– 95.87]
Stroop test dual-task effect (s)	−138.67 [−161.32 to −116.03]	−144.58 [−167.18 to −121.99]
Stroop B&W in *quasi*-static standing posture (s)	74.25 [67.27–81.24]	81.09 [74.09–88.10]
Stroop Color in *quasi*-static standing posture (s)	74.27 [67.79–80.76]	81.10 [74.60–87.59]
Stroop test dual-task effect in *quasi*-static standing posture (%)**	−108.60 [−127.26 to −89.94]	−123.95 [−142.61 to −105.29]

**Notes:**

*((Timed up & Go cognitive version − Timed up & Go conventional version)/Timed up & Go conventional version) × 100.

**((Stroop Color Effect in *quasi*-static standing posture − Stroop Black & White Effect in *quasi*-static standing posture)/Stroop Black & White Effect in *quasi*-static standing posture) × 100.

EG, Experiemental group; CG, Control group; SBER, Static balance with exteroceptive regulation; SBIR, Static balance with interoceptive regulation; DBER, Dynamic balance with exteroceptive regulation; DBIR, Dynamic balance with interoceptive regulation; SBEO, Stable base with eyes open; SBEC, Stable base with eyes closed; SBDO, Stable base with partial vision deprivation (dome); UBEO, Unstable base with eyes open; UBEC, Unstable base with eyes closed; UBDO, Unstable base with partial vision deprivation (dome); Total CTSIB, Total score of the Clinical Test of Sensory Interaction on Balance; 5TSTS, five times sit-to-stand time; FFRT, Forward functional reach test; SRT, sit and rise from the floor test; TMT-A, Trail making test (numerical sequence); TMT-B, Trail making test (alphanumeric sequence); B&W, Black and white.

No significant interaction or group effect was observed in this trial. However, significant time-effect differences were achieved for both training protocols. The mean change (MC) and CI 95% for all time-effect comparisons are shown in Table 2. All significant MC with its respective CI 95% described in the following text and viewed in figures are bolded in Table 2.

**Table 2:** Time effect (mean change, MC) and 95% confidence interval for comparisons between T1 to T2, T2 to T3, and T1 to T3 regarding gait performance, functional mobility, static and dynamic body balance, and cognition function, regardless of the group.

Variable	T1 to T2		T2 to T3		T1 to T3	
	MC	CI 95%	MC	CI 95%	MC	CI 95%
Simultaneous dual task						
Gait speed (m/s)	0.013	[−0,038 to 0.064]	0.026	[−0.024 to 0.077]	0.039	[−0.014 to 0.092]
Gait variability (%)	−0.209	[−0.514 to 0.095]	−0.121	[−0.425 to 0.184]	−0.330	[−0.668 to 0.008]
Gait cycle duration (%)	−0.011	[−0.032 to 0.011]	0.000	[−0.022 to 0.021]	−0.011	[−0.036 to 0.013]
Cadence (steps/min)	1.387	[−0.989 to 3.762]	0.214	[−2.161 to 2.589]	1.601	[−1.038 to 4.240]
Stride length (m)	−0.002	[−0.032 to 0.028]	0.022	[−0.008 to 0.052]	0.020	[−0.014 to 0.054]
Stance (%)	−0.017	[−0.537 to 0.503]	−0.367	[−0.886 to 0.153]	−0.384	[−0.806 to 0.039]
Swing (%)	0.073	[−0.438 to 0.583]	0.411	[−0.099 to 0.922]	**0.484**	[0.088–0.880]
Double support (%)	−0.601	[−1.440 to 0.239]	−0.248	[−1.087 to 0.592]	**−0.849**	[−1.601 to −0.096]
Minimum toe clearance (cm)	**−0.006**	[−0.009 to −0.004]	0.001	[−0.002 to 0.003]	**−0.006**	[−0.008 to −0.003]
Alternating Task						
Gait speed (m/s)	0.005	[−0.034 to 0.044]	0.011	[−0.028 to 0.050]	0.016	[−0.029 to 0.062]
Gait variability (%)	−0.213	[−0.534 to 0.109]	0.028	[−0.294 to 0.349]	−0.185	[−0.501 to 0.131]
Gait cycle duration (%)	−0.008	[−0.030 to 0.014]	0.019	[−0.003 to 0.041]	0.011	[−0.014 to 0.036]
Cadence (steps/min)	1.501	[−0.771 to 3.774]	−0.130	[−2.402 to 2.143]	1.372	[−1.255 to 3.999]
Stride length (m)	−0.007	[−0.037 to 0.023]	0.010	[−0.020 to 0.040]	0.003	[−0.027 to 0.034]
Stance (%)	−0.025	[−0.525 to 0.475]	−0.379	[−0.878 to 0.121]	−0.404	[−0.865 to 0.057]
Swing (%)	−0.010	[−0.731 to 0.711]	**0.986**	[0.265–1.707]	**0.977**	[0.205–1.748]
Double support (%)	−0.439	[−1.210 to 0.333]	−0.707	[−1.478 to 0.065]	**−1.145**	[−1.912 to −0.379]
Minimum toe clearance (cm)	**−0.003**	[−0.006 to −0.001]	0.001	[−0.002 to 0.003]	**−0.003**	[−0.005 to 0.000]
Single Task						
Gait speed (m/s)	−0.018	[−0.065 to 0.029]	−0.025	[−0.072 to 0.022]	−0.042	[−0.092 to 0.008]
Gait variability (%)	−0.097	[−0.350 to 0.156]	0.079	[−0.174 to 0.332]	−0.018	[−0.281 to 0.246]
Gait cycle duration (%)	0.001	[−0.020 to 0.022]	**0.024**	[0.003**–**0.045]	**0.025**	[0.001–0.049]
Cadence (steps/min)	0.574	[−1.849 to 2.998]	−1.683	[−4.107 to 0.741]	−1.108	[−3.898 to 1.682]
Stride length (m)	−0.016	[−0.049 to 0.017)	0.007	[−0.026 to 0.040)	−0.009	[−0.043 to 0.025)
Stance (%)	0.149	[−0.391 to 0.689]	−0.230	[−0.770 to 0.310]	−0.081	[−0.615 to 0.453]
Swing (%)	−0.074	[−0.609 to 0.461]	0.061	[−0.475 to 0.596]	−0.013	[−0.513 to 0.487]
Double support (%)	−0.115	[−1.043 to 0.813]	−0.250	[−1.178 to 0.678]	−0.365	[−1.251 to 0.521]
Minimum toe clearance (cm)	**−0.006**	[−0.009 to −0.003]	0.000	[−0.002 to 0.003]	**−0.006**	[−0.009 to −0.003]
Timed up & Go (conventional) (s)	**−0.738**	[−1.238 to −0.239]	0.432	[−0.067 to 0.931]	−0.307	[−0.067 to 0.931]
Timed up & Go (cognitive) (s)	**−2.191**	[−3.034 to −1.347]	0.544	[−0.300 to 1.387]	**−1.647**	[−2.534 to −0.760]
TUG dual-task effect* (%)	**13.500**	[7.168–19.831]	−0.082	[−6.414 to 6.249]	**13.417**	[6.330–20.505]
SBER (1 to 3)	**0.610**	[0.218–1.003]	**−0.710**	[−1.103 to −0.318]	−0.100	[−0.531 to 0.331]
SBIR (1 to 3)	0.266	[−0.129 to 0.662]	**−0.766**	[−1.162 to −0.371]	**−0.500**	[−0.936 to −0.064]
DBER (1 to 4)	**0.231**	[0.072–0.391]	**−0.298**	[−0.458 to −0.139]	−0.067	[−0.246 to 0.113]
DBIR (1 to 4)	**0.449**	[0.126–0.772]	**−0.449**	[−0.772 to −0.126]	0.139	[−0.344 to 0.344]
SSEO (1 to 4)	**−0.591**	[−0.741 to −0.442]	0.088	[−0.062 to 0.237]	**−0.504**	[−0.646 to −0.361]
SSEC (1 to 4)	**−0.365**	[−0.530 to −0.199]	0.041	[−0.125 to 0.206]	**−0.324**	[−0.459 to −0.189]
SSDO (1 to 4)	**−0.484**	[−0.634 to −0.334]	−0.086	[−0.236 to 0.064]	**−0.570**	[−0.711 to −0.428]
USEO (1 to 4)	−0.060	[−0.152 to 0.031]	−0.003	[−0.090 to 0.083]	−0.064	[−0.139 to 0.011]
USEC (1 to 4)	**−0.594**	[−0.753 to −0.434]	**0.386**	[0.226–0.545]	**−0.208**	[−0.388 to −0.028]
USDO (1 to 4)	**−0.538**	[−0.742 to −0.335]	0.043	[−0.161 to 0.246]	**−0.495**	[−0.721 to −0.270]
Total CTSIB (0 to 24)	**−4.800**	[−6.299 to −3.301]	**−2.167**	[−3.666 to −0.668]	**−6.967**	[−8.676 to −5.257]
5TSTS (s)	**−4.439**	[−6.707 to −2.172]	0.863	[−1.404 to 3.130]	**−3.576**	[−6.014 to −1.138]
FFRT (m)	0.102	[−1.078 to 1.281]	**−1.460**	[−2.639 to −0.281]	−1.358	[−2.752 to 0.036]
SRT (0 to 10)	**0.622**	[0.144–1.100]	0.150	[−0.328 to 0.628]	**0.772**	[0.258–1.286]
TMT-A (s)	**−15.761**	[−28.485 to −3.037]	4.209	[−8.515 to 16.933]	**−11.552**	[−18.741 to −4.362]
TMT-B (s)	**−41.687**	[−64.649 to −18.726]	1.803	[−21.159 to 24.765]	**−39.885**	[−66.726 to −13.043]
TMT (A/B) dual-task effect (s)	8.634	[−34.957 to 52.225]	1.382	[−42.209 to 44.972]	10.016	[−31.697 to 51.729]
Stroop B&W (s)	**−2.841**	[−4.545 to −1.138]	−0.995	[−2.698 to 0.708]	**−3.836**	[−6.086 to −1.587]
Stroop Color (s)	**−21.144**	[−28.537 to −13.750]	−2.526	[−9.919 to 4.867]	**−23.670**	[−31.746 to −15.593]
Stroop test dual-task effect (s)	**44.477**	[24.757–64.198]	1.731	[−17.989 to 1.451]	**46.208**	[25.824–66.592]
Stroop B&W in *quasi*-static standing posture (s)	**−6.229**	[−10.044 to −2.415]	0.457	[−3.357 to 4.272]	**−5.772**	[−10.117 to −1.428]
Stroop Color in *quasi*-static standing posture (s)	**−11.333**	[−18.109 to −4.557]	−1.793	[−8.569 to 4.983]	**−13.126**	[−20.350 to −5.902]
Stroop test dual-task effect in *quasi*-static standing posture (%)**	12.992	[−3.634 to 29.617]	−0.986	[−17.611 to 15.640]	12.006	[−5.782 to 29.794]

**Notes:**

* ((Timed up & Go cognitive version − Timed up & Go conventional version)/Timed up & Go conventional version) × 100.

** ((Stroop Color Effect in quasi-static standing posture − Stroop Black & White Effect in quasi-static standing posture)/Stroop Black & White Effect in quasi-static standing posture) × 100.

Bold mean change (MC) shows significant differences; T1. baseline; T2. intervention end; T3. A total of 24 weeks post-intervention; SBER, Static balance with exteroceptive regulation; SBIR, Static balance with interoceptive regulation; DBER, Dynamic balance with exteroceptive regulation; DBIR, Dynamic balance with interoceptive regulation; SSEO, Stable surface with eyes open; SSEC, Stable surface with eyes closed; SSDO, Stable surface with partial vision deprivation (dome); USEO, Unstable surface with eyes open; USEC, Unstable surface with eyes closed; USDO, Unstable surface with partial vision deprivation (dome); Total CTSIB, Total score of the Clinical Test of Sensory Interaction on Balance; 5TSTS, five times sit-to-stand time; FFRT, Forward functional reach test; SRT, sit and rise from the floor test; TMT-A, Trail making test (numerical sequence); TMT-B, Trail making test (alphanumeric sequence); B&W, Black and white.

**Figure 3:**
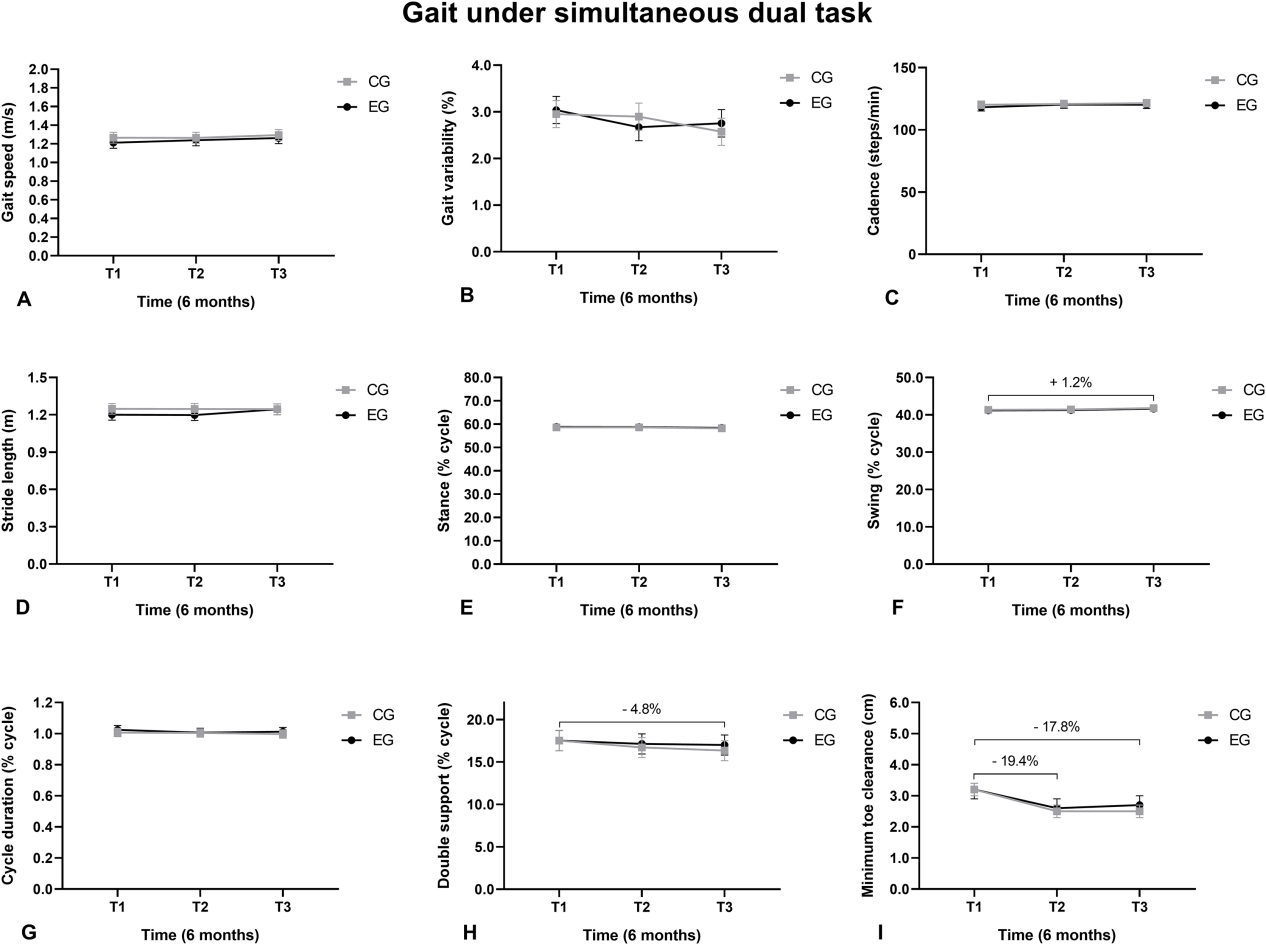
Walking under a simultaneous dual task. (A) Gait speed; (B) Gait variability; (C) Cadence; (D) Stride length; (E) Support phase; (F) Balance phase; (G) Cycle duration; (H) Double support; (I) Minimum toe clearance.

The gait swing phase duration under SDT increased from T1 to T3 (Fig. 3F), the double support phase reduced from T1 to T3 (Fig. 3H), and the minimum toes height reduced from T1 to T2 and from T1 to T3 (Fig. 3I).

**Figure 4:**
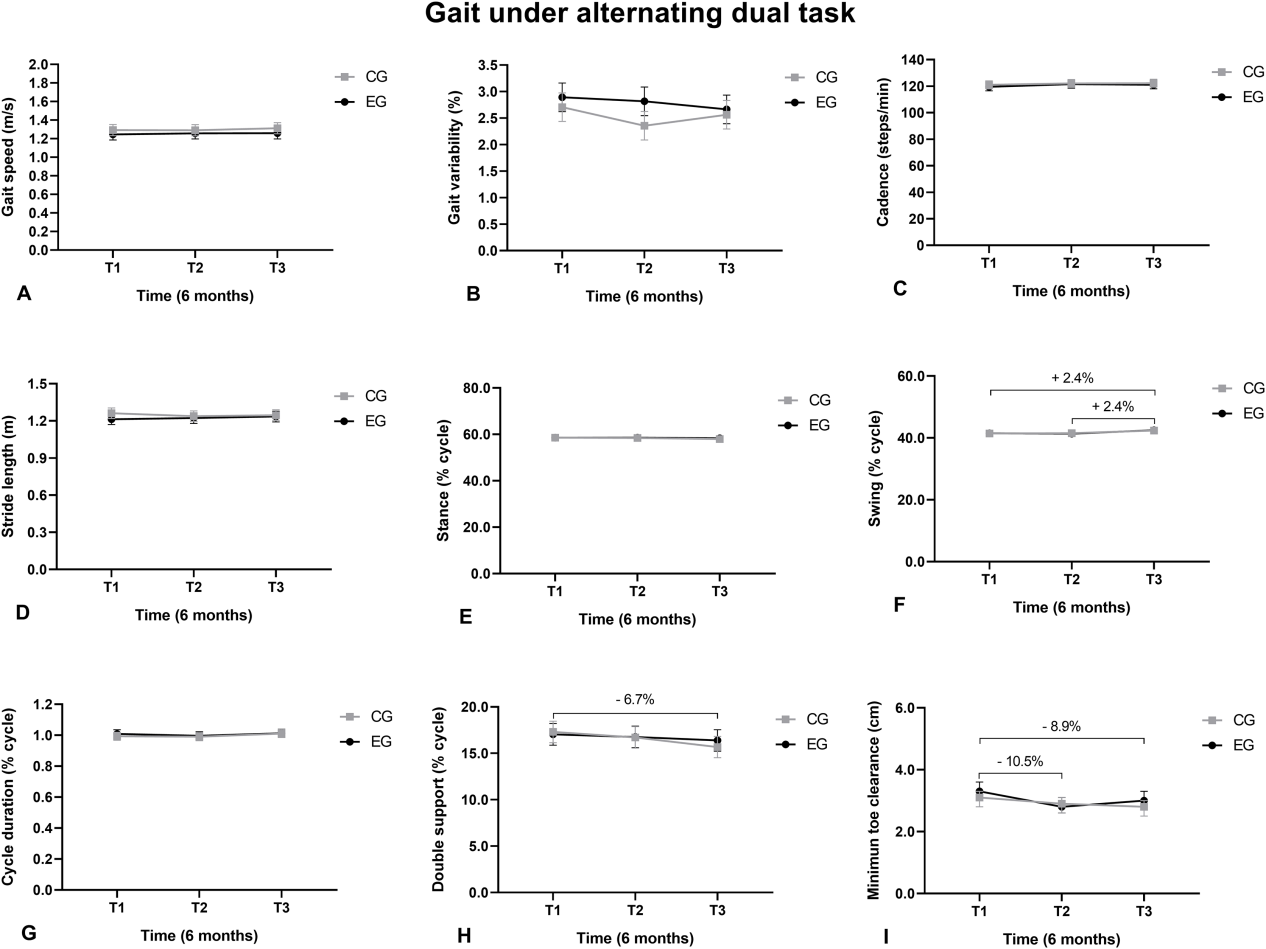
Walking under an alternating task. (A) Gait speed; (B) Gait variability; (C) Cadence; (D) Stride length; (E) Stance; (F) Swing; (G) Cycle duration; (H) Double support; (I) Minimum toe clearance.

The swing phase duration under AT increased from T1 to T3 and from T2 to T3 (Fig. 4F). The double support duration reduced from T1 to T3 (Fig. 4H). The MTC reduced from T1 to T2 and from T1 to T3 (Fig. 4I).

**Figure 5:**
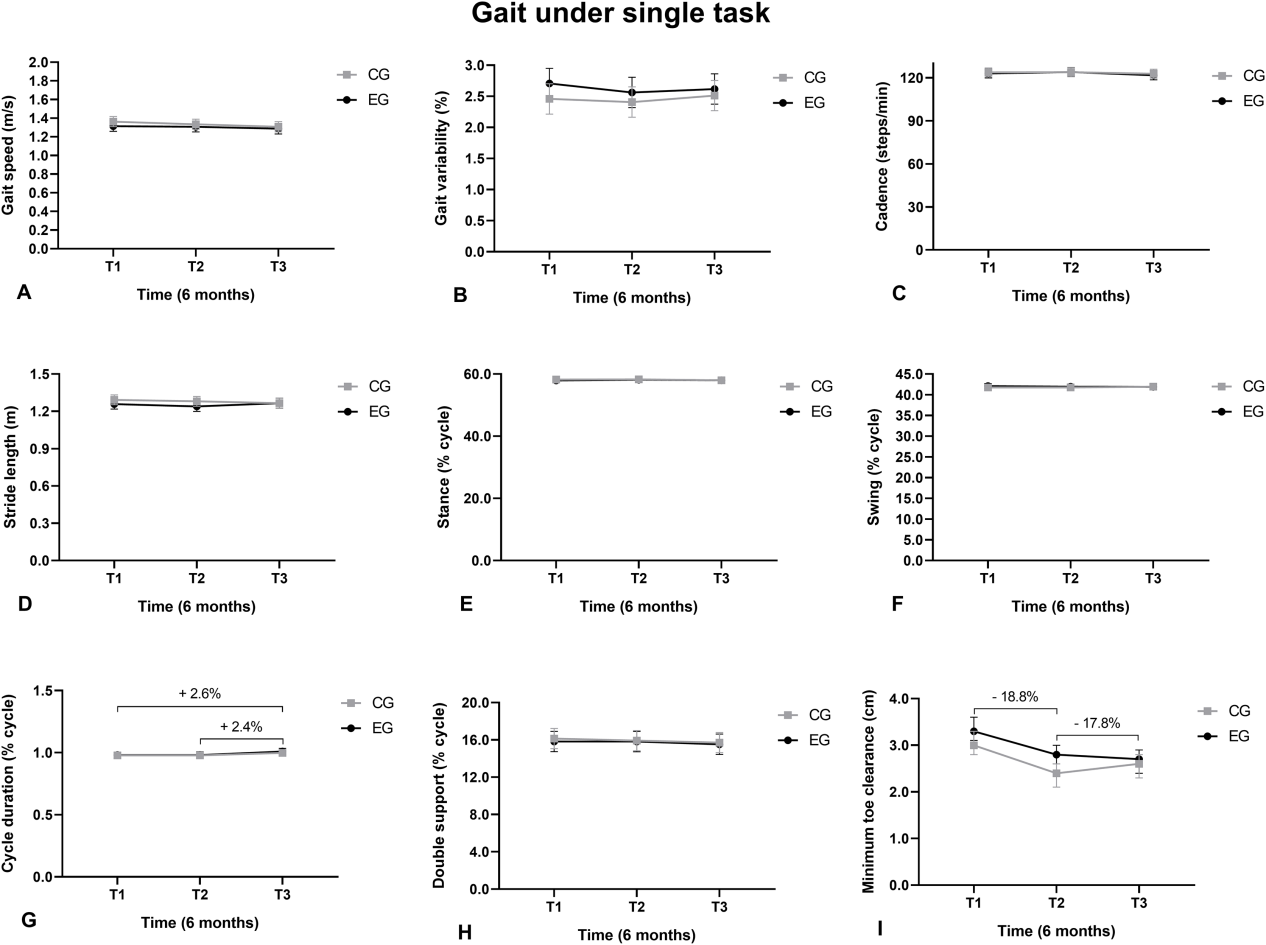
Walking under a single task. (A) Gait speed; (B) gait variability; (C) cadence; (D) stride length; (E) support phase; (F) balance phase; (G) cycle duration; (H) double support; (I) minimum toe clearance.

The gait cycle length under SMT increased from T1 to T3 and from T2 to T3. The MTC reduced from T1 to T2 and from T1 to T3 (Fig. 5I).

**Figure 6:**
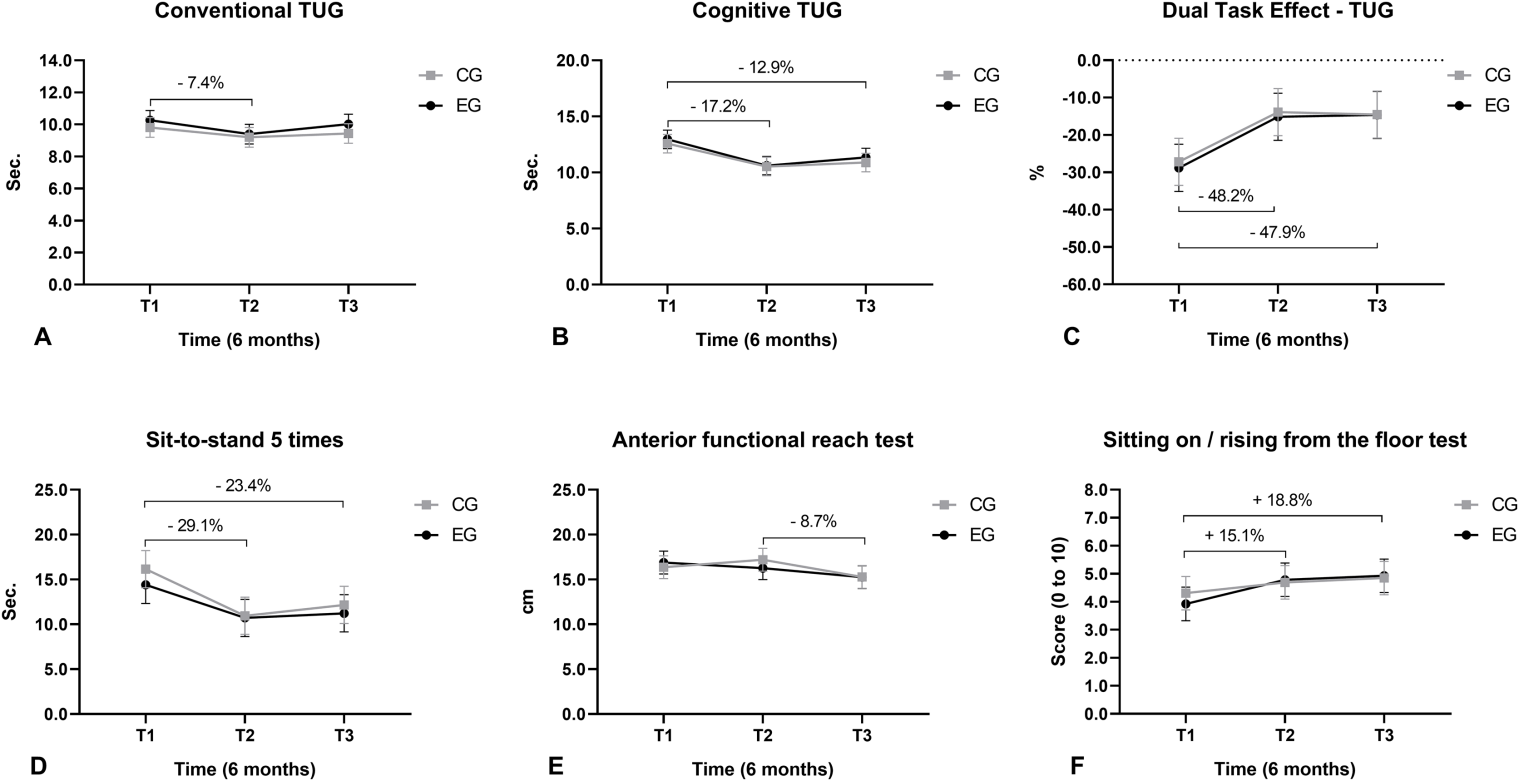
Mobility function. (A) Conventional TUG; (B) cognitive TUG; (C) dual-task effect for TUG; (D) get-up and sit five times; (E) forward functional reach test; (F) sit and get up off the floor.

Regarding mobility, we observed that the execution time of the conventional TUG reduced from T1 to T2 (Fig. 6A) and the execution time of the cognitive TUG reduced from T1 to T2 and from T1 to T3 (Fig. 6B). The dual-task cost (negative values) when performing the TUG (cognitive *vs*. conventional) reduced from T1 to T2 and from T1 to T3 (Fig. 6C). The time taken to perform the 5TSTS reduced from T1 to T2 and from T1 to T3 (Fig. 6D). The FFRT distance decreased from T2 to T3 (Fig. 6E). The scores for the sitting and rising from the floor test (SRT) increased from T1 to T2 and from T1 to T3 (Fig. 6F).

**Figure 7:**
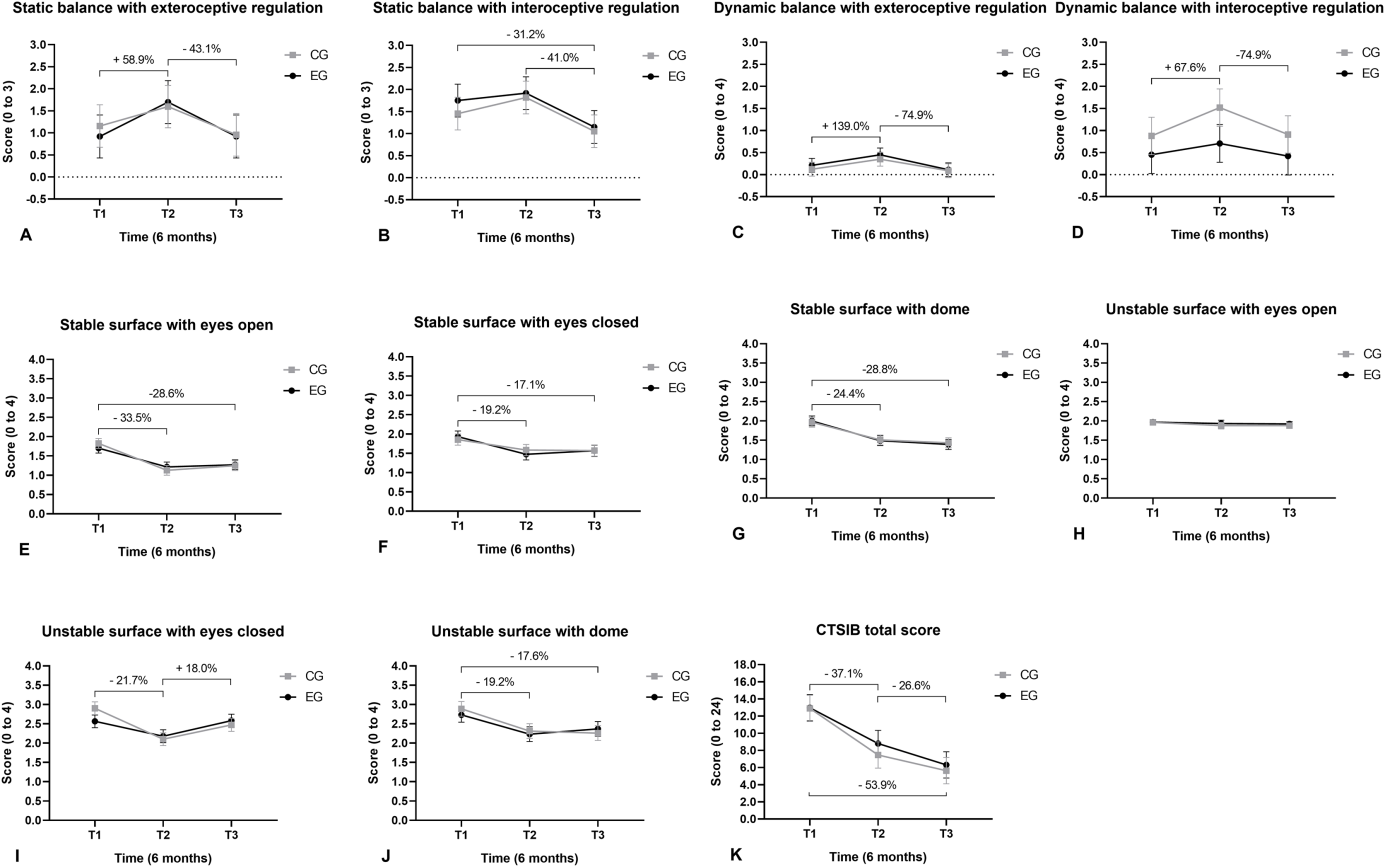
Balance. (A) Static balance with exteroceptive regulation; (B) static balance with interoceptive regulation; (C) dynamic balance with exteroceptive regulation; (D) dynamic balance with interoceptive regulation; (E) static balance on a stable surface with eyes open; (F) static balance on a stable surface with eyes closed; (G) static balance on a stable surface with visual conflict; (H) static balance on an unstable surface with eyes open; (I) static balance on an unstable surface with eyes closed; (J) static balance on an unstable surface with visual conflict; (K) total CTSIB score.

Static postural balance with exteroceptive regulation increased (improved) from T1 to T2 and reduced from T2 to T3 (Fig. 7A). Static balance with interoceptive regulation reduced from T2 to T3 and from T1 to T3 (Fig. 7B). The dynamic postural balance with exteroceptive regulation increased from T1 to T2 and reduced from T2 to T3 (Fig. 7C). The dynamic postural balance with interoceptive regulation increased from T1 to T2 and reduced from T2 to T3 (Fig. 7D). Body sway in the upright quiet standing posture for the CTSIB scores reduced (improved) on a stable surface with eyes open, from T1 to T2 and from T1 to T3 (Fig. 7E). Body oscillation on a stable surface with eyes closed reduced from T1 to T2 and from T1 to T3 (Fig. 7F). Body sway on a stable surface with visual conflict reduced from T1 to T2 and from T1 to T3 (Fig. 7G). Body oscillation on an unstable surface with eyes closed reduced from T1 to T2 and increased from T2 to T3 (Fig. 7I). Body sway on an unstable surface with visual conflict was reduced from T1 to T2 and from T1 to T3 (Fig. 7J). The total CTSIB score for body sway in upright quiet standing posture decreased (improved) from T1 to T2 and from T2 to T3 (Fig. 7K).

**Figure 8:**
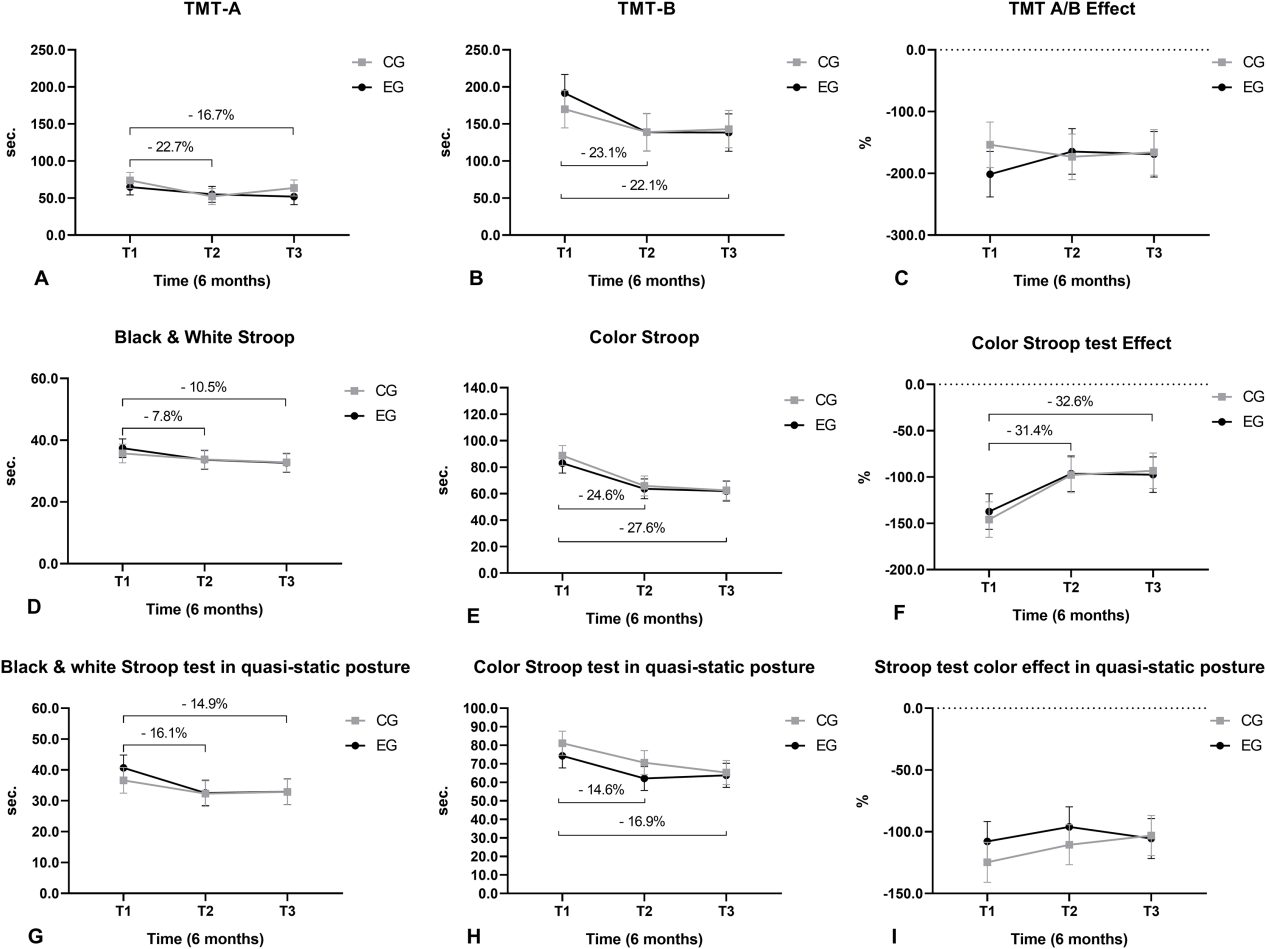
Cognition function. (A) TMT-A; (B) TMT-B; (C) dual-task effect concerning TMT A/B; (D) Black and white Stroop test; (E) Color Stroop test; (F) effect of the Black &White and color Stroop test; (G) Black & White test in *quasi*-static; (H) Color Stroop test in *quasi*-static; (I) effect of the Black & White and colored Stroop test *quasi*-static.

The execution time of TMT-A reduced from T1 to T2 and from T1 to T3 (Fig. 8A). The execution time of the TMT-B reduced from T1 to T2 and from T1 to T3 (Fig. 8B). The execution time of the monochromatic (black and white) version of the Stroop test reduced from T1 to T2 and from T1 to T3 (Fig. 8D). The execution time of the colored version of the Stroop test reduced from T1 to T2 and from T1 to T3 (Fig. 8E). The cost (negative values) of executing the color version to the monochrome version of the Stroop test reduced from T1 to T2 and from T1 to T3 (Fig. 8F). The execution time of the monochromatic version of the Stroop test in upright quiet standing posture reduced from T1 to T2 and from T1 to T3 (Fig. 8G). The execution time of the color version of the Stroop test in upright quiet standing posture reduced from T1 to T2 and from T1 to T3 (Fig. 8H).

## Discussion

We aimed to compare the effects of a mixed dual-task protocol training in which participants underwent initially SMT and SDT interchangeably and progressed strictly with SDT regarding a control protocol in which participants only performed SMT and SDT activities alternately. Contrary to what we expected, participants of the experimental protocol did not improve their gait performance, functional mobility, static and dynamic body balance, and cognition function compared to the participants of the control protocol over 24 weeks. However, the performance of both groups improved in some functional outcomes after 24 weeks. Also, the gains obtained after the 24-week follow-up post-intervention period for both groups were not maintained for the following 24 weeks post-intervention.

Although the functional balance, mobility, and cognition of participants in both groups have improved, the gait speed under SDT (primary outcome) and most of the gait spatiotemporal parameters did not change, except for the MTC. This result can be explained by the principle of training specificity, in which it is highlighted that the adaptations achieved during training are strongly associated with the mode, frequency, and duration of the proposed exercise (Hawley, 2008). Specific demands must be incorporated to ensure optimal performance (Reilly, Morris & Whyte, 2009). The closer the training routine to the desired result, the better the result/response (Hawley, 2008). A training program needs to emphasize the systems which are involved in carrying out an activity to obtain specific training adaptations (Reilly, Morris & Whyte, 2009). Thus, we believe that the participants of both groups may not have been specifically stimulated by the proposed mixed dual-task protocol to improve walking speed and other gait parameters. On the other hand, both groups obtained improvement in mobility (assessed by TUG), interoceptive and exteroceptive function (assessed by the postural balance test), static and dynamic body balance (assessed by the CTSIB), cognitive performance (assessed by the effects of dual task regarding the TUG cognitive, TMT and Stroop test) and lower limb performance (assessed by 5TSTS test). We suppose we would have to increase the task specificity, intensity, and volume of training to increase the walking speed and other biomechanical variables to obtain significant between-group differences over time with the proposed protocol. The great diversity of training foci adopted in our intervention may have imposed too many stimuli on the participants which, in the end, did not contribute to increase the gait speed itself.

Another explanation for our results concerning gait speed is based on the study by Hortobagyi et al. (2015) that concluded that gait speed was higher after resistance and multimodal training than coordination exercises for this population. By considering the characteristics of our experimental protocols, a longer intervention period may be needed to achieve improvement in the gait speed for community-dwelling older adults whose baseline magnitude of this outcome was already functionally good (equal or higher than 1 m/s).

An almost 20% reduction in the MTC in all gait conditions caught our attention, as we expected that this outcome would increase and not decrease from the protocol training. We know that the lower the minimum toe clearance, the greater the risk of falls, as this gait parameter is strongly linked to unintentional contact of the foot with the floor (Killeen et al., 2017). The motor control mechanisms acting during a single gait can enable prioritizing critical parameters such as regulation of the minimum toe clearance, rather than others such as speed (Hamacher et al., 2016). On the other hand, the literature highlights that walking is not an automated task and requires attention mainly in more complex and demanding situations, such as those under SDT. In these situations, there is a reduction in the execution of one or both activities (Brustio et al., 2017). This explanation supports the results of our study since the increased cognitive demand imposed during the dual-task training protocols may have led to prioritizing the participants’ cognitive performance. Regardless of the dual-task protocol, an increase in cognitive demand may have resulted in an increase in gait ‘automatism’ and, as a result, a lower MTC. According to Hamacher et al. (2019), by adopting a more automated gait pattern, a reduced MTC is observed in both young individuals and older adults. Thus, it is expected that cognitive-motor functioning will deteriorate in complex situations as there is less cognitive and motor reserve available to perform the required task (Wollesen & Voelcker-Rehage, 2014).

Our results regarding cognitive performance support the points raised before even more. Both groups improved sustained and alternating attention, mental flexibility, visual processing speed, and manual motor function, as assessed by versions A and B of TMT. Participants from both groups also showed improvement in selective attention and aspects of executive functions, such as cognitive flexibility and susceptibility to interference. Although these are very positive results for cognitive reserve, they may have been the cause of the increase in gait automatism and consequent reduction of the MTC after the 24-week intervention.

In addition to cognitive function, both protocols improved static and dynamic balance. Physical exercise induces structural plasticity in the human brain, which results in an improvement in cognitive functions which are directly related to balance, such as visual and vestibular functions (Rogge et al., 2018). The decrease in balance is associated with reduced physical functioning, which may compromise the performance of some functional activities of daily living. Thus, from a motor control point of view, we realized that both protocols managed to have interesting effects in improving both forms of body balance. Unlike the high gait complexity, we can see that cognitive gain did not reduce the participant’s ability to maintain body balance, but the opposite.

The higher proportion of older women in this study must be addressed. The multifaceted phenomenon known as feminization of aging, in which more and more women are accounted for in the older population, especially in Brazil (Cepellos, 2021), can explain this imbalance regarding the proportion of women and men. Literature has shown that older women consult their general practitioner more often than men (Hunt et al., 2011). Thus, the feminization of aging can explain the higher interest of community-dwelling older women to participate in this clinical trial. Furthermore, the results of two large Brazilian studies (Elias-Filho et al., 2019; Pimentel et al., 2018) showed a higher ratio of older women with a fall history. Like the studies above-mentioned, the sex ratio of participants in our study corroborates social and health realities, as we found that there are more women than men interested in participating in clinical trials to improve their health.

The strengths of this study include (i) the rigorous randomized controlled trial methodology; (ii) the adoption of a more robust statistical model (GLMM) to minimize the negative impact of missing data, although it was relatively compensated by the sample size calculation that included 20% of expected dropouts; (iii) the relatively long-term intervention and follow-up periods which took place over 24 weeks each. In this study we have chosen to investigate a novel mixed dual task protocol. We also chose on maintaining a longer intervention period (6 months). However, adding some physical multicomponent throughout a short-term intervention period can be considered in future studies regarding dual-task protocol training.

Some limitations need to be considered as well. Our results should be considered only for older adults with a low level of cognitive impairment and gait speed equal to or higher than 1 m/s, and participants with no recurrence of falls. The results may not be the same in those individuals with recurrent falls and even those with post-fall syndrome.

## Conclusion

Our results suggest the proposed mixed dual-task protocol training in which participants initially underwent SMT and SDT interspersing and progressed strictly with SDT was not superior to the control protocol in which participants only performed SMT and SDT activities interchangeably. Therefore, both protocols can be applied in clinical settings to enhance gait, balance, and cognitive function in community-dwelling older adults.

## Supplemental Information

10.7717/peerj.15030/supp-1 Supplemental Information 1 CONSORT Checklist.

10.7717/peerj.15030/supp-2 Supplemental Information 2 Raw data. All the gait, cognition and postural balance outcomes of the participants acquired at time 1, 2 and 3.

## Additional Information and Declarations

### Data Availability

The following information was supplied regarding data availability: The raw measurements are available in the Supplemental Files.
